# The evolution of acute lymphoblastic leukemia research and therapy at MD Anderson over four decades

**DOI:** 10.1186/s13045-023-01409-5

**Published:** 2023-03-16

**Authors:** Elias Jabbour, Nicholas J. Short, Nitin Jain, Fadi G. Haddad, Mary Alma Welch, Farhad Ravandi, Hagop Kantarjian

**Affiliations:** Department of Leukemia, The University of Texas MD Anderson Cancer Center, 1515 Holcombe Boulevard, Unit 428, Houston, TX 77030 USA

**Keywords:** Blinatumomab, CAR T cell, Chemotherapy-free, Cure, Evolution, Inotuzumab, Ponatinib, Targeted therapies, Tyrosine kinase inhibitors

## Abstract

Progress in the research and therapy of adult acute lymphoblastic leukemia (ALL) is accelerating. This analysis summarizes the data derived from the clinical trials conducted at MD Anderson between 1985 and 2022 across ALL subtypes. In Philadelphia chromosome-positive ALL, the addition of BCR::ABL1 tyrosine kinase inhibitors (TKIs) to intensive chemotherapy since 2000, improved outcomes. More recently, a chemotherapy-free regimen with blinatumomab and ponatinib resulted in a complete molecular remission rate of 85% and an estimated 3-year survival rate of 90%, potentially reducing the role of, and need for allogeneic stem cell transplantation (SCT) in remission. In younger patients with pre-B Philadelphia chromosome-negative ALL, the integration of blinatumomab and inotuzumab into the frontline therapy has improved the estimated 3-year survival rate to 85% across all risk categories. Our future strategy is to evaluate the early integration of both immunotherapy agents, inotuzumab and blinatumomab, with low-dose chemotherapy (dose-dense mini-Hyper-CVD-inotuzumab-blinatumomab) into the frontline setting followed by CAR T cells consolidation in high-risk patients, without any further maintenance therapy. In older patients, using less intensive chemotherapy (mini-Hyper-CVD) in combination with inotuzumab and blinatumomab has improved the 5-year survival rate to 50%. Among patients ≥ 65–70 years, the mortality in complete remission (CR) is still high and is multifactorial (old age, death in CR with infections, development of myelodysplastic syndrome or acute myeloid leukemia). A chemotherapy-free regimen with inotuzumab and blinatumomab is being investigated. The assessment of measurable residual disease (MRD) by next-generation sequencing (NGS) is superior to conventional assays, with early MRD negativity by NGS being associated with the best survival. We anticipate that the future therapy in B-ALL will involve less intensive and shorter chemotherapy regimens in combination with agents targeting CD19 (blinatumomab), CD20, and CD22 (inotuzumab). The optimal timing and use of CAR T cells therapy may be in the setting of minimal disease, and future trials will assess the role of CAR T cells as a consolidation among high-risk patients to replace allogeneic SCT. In summary, the management of ALL has witnessed significant progress during the past four decades. Novel combination regimens including newer-generation BCR::ABL1 TKIs and novel antibodies are questioning the need and duration of intensive chemotherapy and allogeneic SCT.

## Background

The management of adult acute lymphoblastic leukemia (ALL) is evolving rapidly. The classical adult ALL regimens deviated early on from the principles applied in pediatric regimens (shorter maintenance; reliance on allogeneic and autologous stem cell transplantation [SCT] in remission) and resulted in estimated 5-year survival rates of 30% to 35% [1]. In 1992, we developed the hyper-CVAD regimen (fractionated cyclophosphamide, vincristine, doxorubicin, and dexamethasone) in patients with newly diagnosed B-ALL [2]. This is a pediatric-inspired regimen that relegated asparaginase therapy to the consolidation phases (rather than during induction for active disease) in order to reduce the potential asparaginase-associated toxicities. This dose-intensive regimen resulted in improved outcome in adult B-ALL with a complete remission (CR) rate of 90 + % and a 5-year overall survival (OS) rate of 40%. This regimen incorporated central nervous system (CNS) prophylaxis with intrathecal (IT) chemotherapy consisting of cytarabine and methotrexate. It demonstrated the efficacy of IT chemotherapy in combination with systemic chemotherapy in reducing the risk of CNS recurrence, allowing the omission of cranial irradiation and associated adverse events [3, 4]. To further improve outcomes, in 2000, we added rituximab as anti-CD20 therapy to hyper-CVAD based on the findings that CD20 expression (≥ 20%) on B-ALL blasts was associated with worse outcome [5]. The addition of rituximab improved the outcomes in patients with CD20-positive disease with 3-year OS rate of 50% overall and of 75% in younger patients < 60 years [6]. Hyper-CVAD was also combined with BCR::ABL1 tyrosine kinase inhibitors (TKI) in the frontline therapy of Philadelphia chromosome (Ph)-positive ALL and resulted in high CR and complete molecular response (CMR) rates, a higher proportion of patients bridged to allogeneic SCT, and a superior survival. Outcomes steadily improved with each newer generation of TKI, beginning with imatinib, then dasatinib, and most recently ponatinib [7–11].

Since early 2010, antibodies targeting CD19 and CD22 have been evaluated in relapsed/refractory (R/R) B-ALL [12–14], leading to the approval of these agents as monotherapy in the salvage setting [15, 16]. These included blinatumomab, a CD19 targeting bispecific T cell engager (BiTE) approved in 2014, and inotuzumab, a CD22 targeting antibody–drug conjugate, approved in 2017 [15, 16]. Therapeutic strategies have then moved to combination therapies in both the salvage and frontline settings, contributing to the improved survival in newly diagnosed B-ALL.

**Fig. 1:**
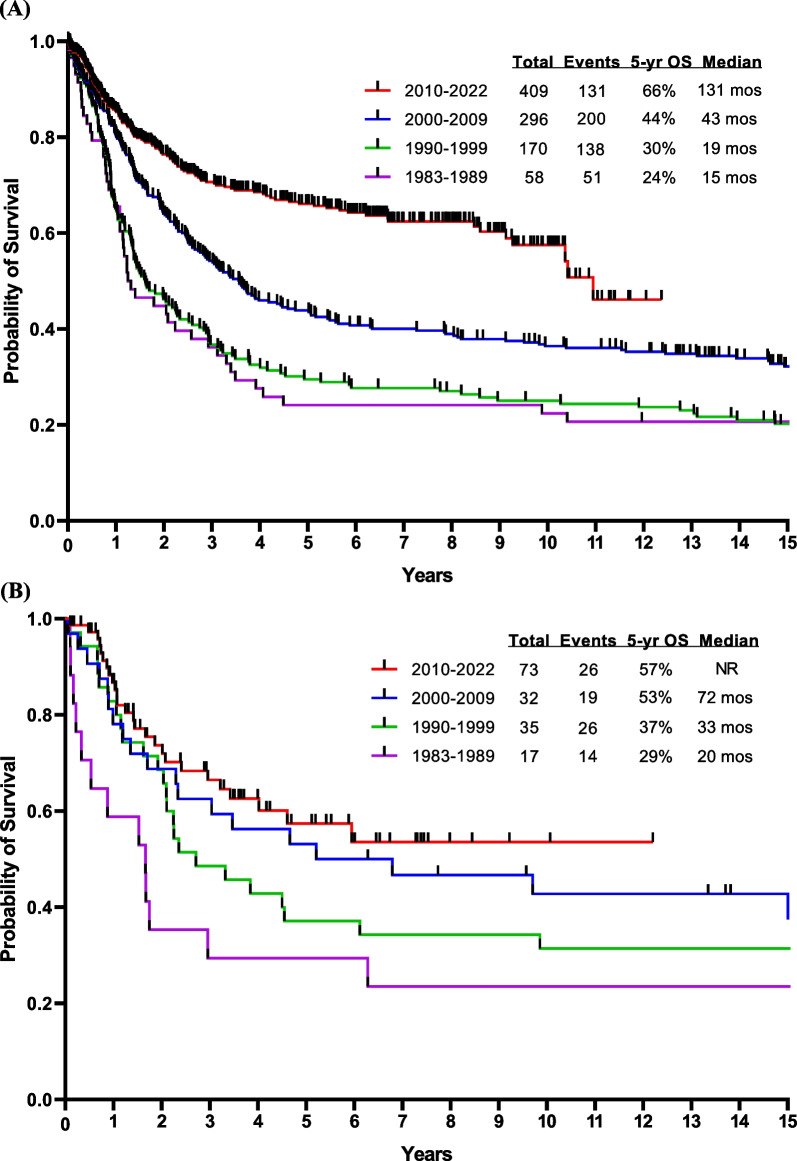
Overall survival by decade at MDACC of (**A**) B-ALL and (**B**) T-ALL. Abbreviations: mos, months; OS, overall survival

Cytogenetics play an important role in independent of molecular abnormalities [17, 18]. High-risk cytogenetic features include *KMT2A* rearrangements, complex cytogenetics, and low hypodiploidy or near haploidy. Low hypodiploidy and complex cytogenetics are also associated with the presence of *TP53* mutations. The presence of *TP53* mutations, CRLF2 overexpression, and *JAK2* mutation are particularly associated with a poor prognosis [19]. However, the introduction of novel agents and targeted antibodies such as blinatumomab and inotuzumab, combined with a better understanding of disease biology, has translated into a better survival [20, 21]. We have recently reported on the results from the Surveillance, Epidemiology, and End Results (SEER) database that show an improvement in survival over the past five decades in adult ALL [22]. Data from MD Anderson show similar survival improvements over the past four decades in both B-ALL (Fig. 1A) and T-ALL (Fig. 1B).

Race and ethnicity have been also shown to impact survival. In the SEER database analysis between 2000 and 2017, Hispanic and African American ethnicity were independent factors associated with worse outcome. The Ph-like ALL phenotype is more prevalent among Hispanic patients, which could explain the worse outcome observed in this population. Among African Americans, the higher incidence of comorbidities (metabolic syndrome and cardiovascular problems) and the poor financial status are the main factors associated with poor outcome [22].

**Fig. 2:**
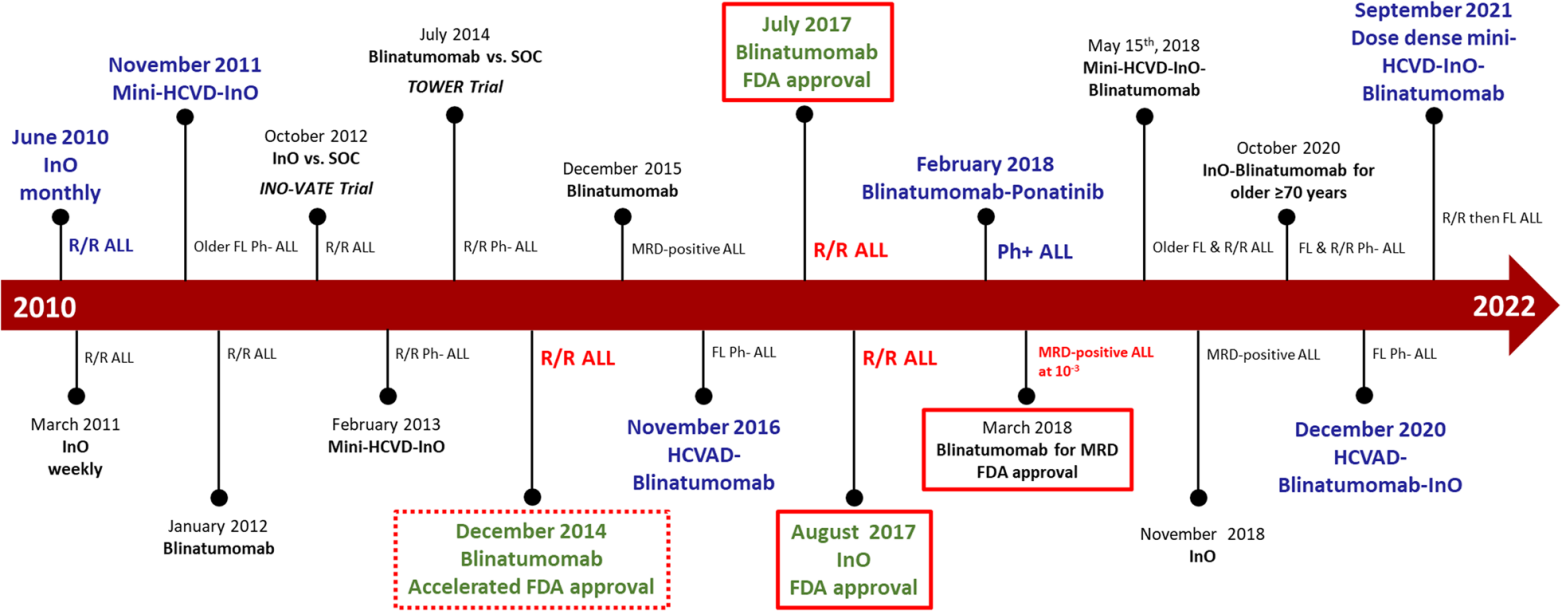
The evolving treatment landscape of B-ALL. Abbreviations: FL, frontline; InO, inotuzumab ozogamicin; MRD, measurable residual disease; Ph, Philadelphia chromosome; R/R, relapsed/refractory; SOC, standard of care

Despite the efforts to develop new therapeutic strategies over the past 10 years and the number of clinical trials conducted, the evolution of ALL therapy and the adoption of new standard regimens remain slow (Fig. 2). Herein, we report the outcomes of patients with ALL treated at MD Anderson over the past four decades and discuss the results in relation to ongoing research and published data from outside our institution in ALL.

We reviewed the data derived from clinical trials conducted at MD Anderson between 1985 and 2022 across the subtypes of ALL. The results of these trials were reported in detail separately. We described the outcomes in these clinical trials including responses, survival, and rates of allogeneic SCT. Outcomes were stratified by time period, disease subtype, and patient age, highlighting the improvement in survival across different groups treated in a single cancer center. We then compared the outcomes to those published in the literature, in a descriptive fashion. The Kaplan–Meier method was used for survival analysis with the log-rank test.

## Main text

### Ph-positive ALL

**Fig. 3:**
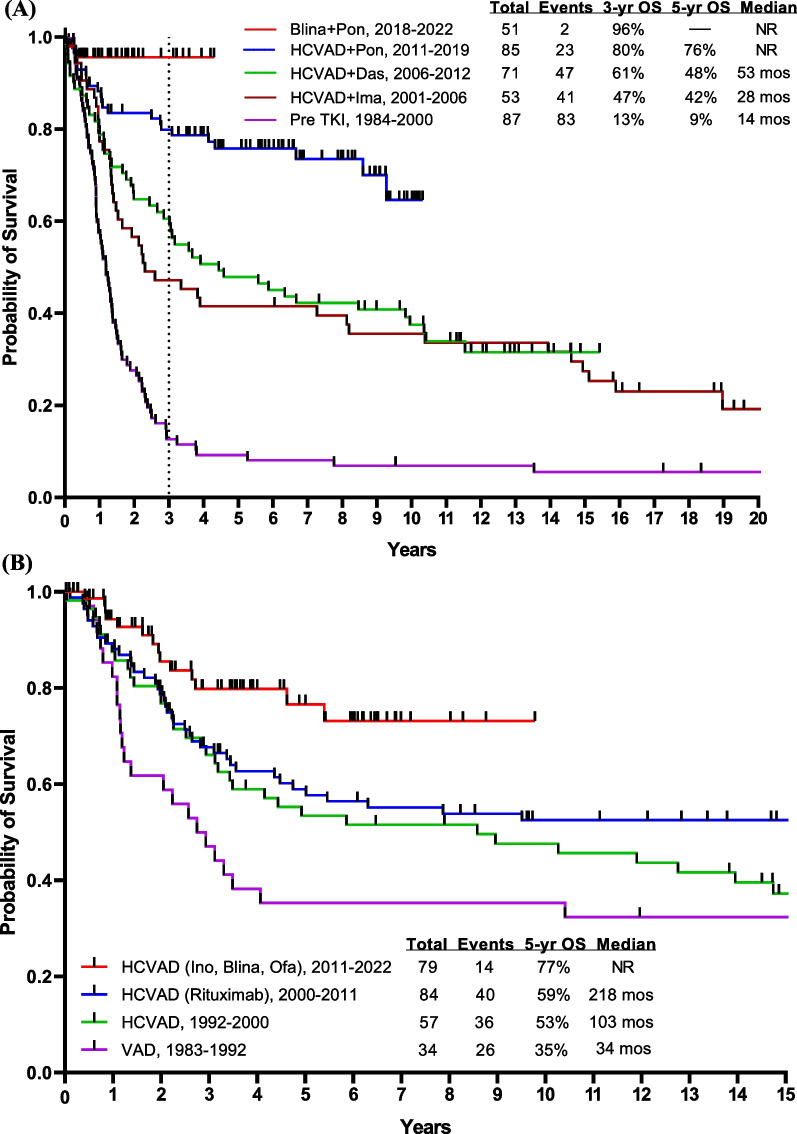

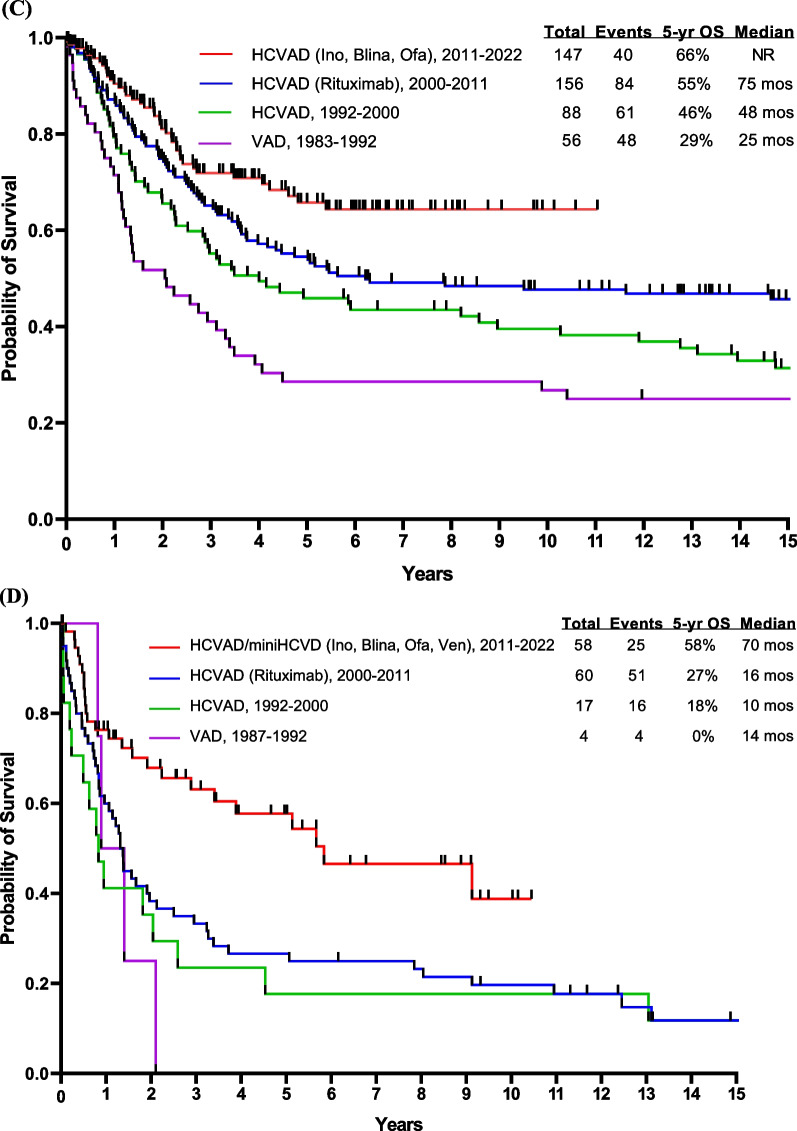
Overall survival by treatment era. **A** Ph-positive ALL, **B** AYA patients having Ph-negative ALL, **C** Ph-negative ALL < 60 years, and **D** Ph-negative ALL ≥ 60 years. Abbreviations: blina, blinatumomab; das, dasatinib; HCVAD, Hyper-CVAD regimen; ima, imatinib; ino; inotuzumab; NR, not reached; ofa, ofatumumab; OS, overall survival; pon, ponatinib; TKI, tyrosine kinase inhibitor; VAD, vincristine, adriamycin, dexamethasone; ven, venetoclax

Before 2000, the year when BCR::ABL1 TKIs were introduced into the treatment of Ph-positive ALL, the outcome of such patients was very poor. These patients had a 5-year OS rate of < 10% with intensive chemotherapy and 30–40% if they were able to undergo allogeneic SCT in first CR [23–25]. With the advent of regimens that combined chemotherapy with BCR::ABL1 TKIs (imatinib in 2000, dasatinib in 2006, ponatinib in 2010), the 5-year OS rates improved from < 10% to 50% (Fig. 3A). The combination of hyper-CVAD and imatinib resulted in a CR rate of > 90% and a long-term OS rate of 40% [10]. The subsequent hyper-CVAD combination with dasatinib resulted in a CR rate of 96% and a 5-year OS rate of 46% [26].

The T315I mutation of the ABL1 kinase domain is highly resistant to imatinib and to second-generation TKIs and is detected in up to 75% of patients relapsing after first- and second-generation TKIs, with a higher incidence in older patients compared with younger adults [27, 28]. Ponatinib, a potent pan-BCR::ABL1 inhibitor, suppresses the T315I mutation and eradicates molecular measurable residual disease (MRD) [29]. The combination of hyper-CVAD plus ponatinib investigated in 86 patients (median age, 46 years) with newly diagnosed Ph-positive ALL, produced a CMR rate of 74% at 3 months and a cumulative CMR rate of 86% [30, 31]. After a median follow-up of 80 months, the 6-year OS rate for the entire cohort was 75%. An 8-month landmark analysis showed a trend for better OS in patients who did not undergo allogeneic SCT in the first remission. In the original protocol, ponatinib was administered at 45 mg daily [29]. After two patients had fatal ponatinib-associated cardiovascular toxicities, the protocol was amended to allow for a response-adjusted dosing of ponatinib (i.e., 45 mg in Course 1, 30 mg in CR, and 15 mg in CMR) [30]. This strategy reduced the risk of cardiovascular events, making the treatment more manageable; no further ponatinib-related mortality has been observed since then. For CNS prophylaxis, 12 IT chemotherapy doses were given, which reduced the rate of CNS relapses compared with 8 IT doses (6-year CNS relapse rate, 0% versus 13%) [32, 33]. In a propensity-matched score analysis of hyper-CVAD plus ponatinib and hyper-CVAD plus dasatinib, ponatinib was associated with higher rates of CMR at 3 months and superior OS [34]. A meta-analysis of 26 clinical trials also confirmed the superiority of ponatinib over earlier-generation TKIs, with higher rates of CMR and survival [35]. Patients who achieve CMR at 3 months had superior OS compared with patients with detectable disease at 3 months (4-year OS rate, 66% versus 36%) [36]. Among patients who achieved CMR at 3 months, ponatinib therapy was independently associated with improved outcomes compared with dasatinib or imatinib, with better progression-free survival (PFS) and OS [37]. Therefore, patients who achieve early CMR at 3 months have an excellent survival, and allogeneic SCT may not be needed [36–38]. This is in contrast to studies done across the USA and Europe, where allogeneic SCT remains a standard of care in first CR.

### Can we reduce or eliminate the need for intensive chemotherapy in Ph-positive ALL?

Several studies from Italy and Europe have reported on the efficacy of TKI-based regimens combined with low-dose chemotherapy or no chemotherapy [39–46]. They showed CMR rates of 20–40%, 3- to 5-year OS rates of 30–40%, and a high incidence of T315I mutations at relapse. Inotuzumab and blinatumomab had shown efficacy in R/R Ph-positive ALL, leading to the use of blinatumomab in combination with TKIs in the frontline setting [47, 48]. Our group reported retrospectively on the safety and efficacy of a chemotherapy-free approach in Ph-positive ALL; a combination of blinatumomab plus TKIs resulted in a CMR rate of 75% and a 1-year OS rate of 73% [49]. The GIMEMA LAL2116 D-ALBA trial treated 63 patients (median age, 54 years) with dasatinib and the addition of blinatumomab 3 months into therapy. Overall, 60% of patients achieved a deep molecular response (41% CMR) after at least 2 courses of blinatumomab [50]. At a median follow-up of 40 months, the estimated 4-year OS and disease-free survival (DFS) rates were 78% and 75%, respectively. Fifty percent of patients received allogeneic SCT in CR. Nine patients relapsed: 4 hematologic relapses, 4 CNS relapses, and 1 nodal relapse [51]. At MD Anderson, we initiated a frontline regimen combining ponatinib and blinatumomab given together during induction, with continuation of blinatumomab for 5 total courses. Ponatinib 30 mg during induction-maintenance was reduced to 15 mg in CMR [52]. Among 44 patients treated in the frontline setting, the rates of CMR after one course and overall were 64% and 85%, respectively. Among 25 patients who had MRD assessment by next-generation sequencing (NGS), 22 (88%) had undetectable disease at a sensitivity level of 10^−6^. The estimated 3-year PFS and OS rates were both 95% (Fig. 3A). Only one patient underwent allogeneic SCT in first remission due to persistently detectable *BCR::ABL1* transcript levels < 0.05%. Thus, the future management of Ph-positive ALL may consist of chemotherapy-free and allogeneic SCT-sparing targeted therapies. Intensive chemotherapy may still be needed in some subsets: *IKZF1*^plus^ (*IKZF1* deletion plus additional genetic aberrations including *CDKN2A*, *CDKN2B*, or *PAX5*), mixed lineage dual lymphoid and myeloid clones, documentation of Philadelphia chromosome detected by FISH in differentiated myeloid cells. The decision of proceeding with allogeneic SCT depends on the early achievement of CMR within the first 3 months of therapy, which was shown to translate into improved OS. Furthermore, the recent introduction of NGS for the detection of MRD allows a more precise discrimination of patients between those who clear or not their leukemia blasts, at a sensitivity of 10^−6^. Patients who achieve MRD negativity by NGS could potentially be cured without the need for an allogeneic SCT and might potentially be candidates for treatment discontinuation [53].

### Ph-negative adult pre-B ALL (age up to 60 years)

Adolescent and young adults (AYA; age 15–39 years) with ALL have traditionally been treated with pediatric-inspired regimens. The CALGB trial showed a 3-year survival rate of 73%. Hyper-CVAD-based therapy (also a pediatric-inspired regimen developed in 1992, and which delays asparaginase to later consolidations) showed equivalent results (Fig. 3B). The addition of the anti-CD20 antibodies, rituximab in 2000 and ofatumumab in 2012, to hyper-CVAD has improved the survival [6]. A propensity-score matching analysis showed ofatumumab to result in better outcomes than rituximab, when added to hyper-CVAD [6, 54–56]. Further improvement may be observed with the use of novel anti-CD20 agents such as bispecific anti-CD3/CD20 T cell engagers, which showed encouraging results in patients with non-Hodgkin lymphomas [57, 58].

A better understanding of the B-ALL biology allows for better stratification of patients and for better subset-oriented therapies. For example, Ph-like ALL was recognized as a provisional entity by the World Health Organization (WHO) in 2016. It includes patients whose gene expression profile is similar to that of Ph-positive ALL but without the t(9;22) chromosomal abnormality [59]. The WHO 2022 classification and the International Consensus Classification recognized Ph-like ALL as having a wide variety of genetic lesions, alterations, and fusions, some of which could have therapeutic implications [60, 61]. There are 2 major subtypes of Ph-like ALL: CRLF2 rearranged and non-CRLF2 rearranged. Flow cytometry and FISH are used to assess CRLF2 status in all Ph-negative B-ALL patients. Those who no rearrangement is present and/or CRLF2 protein expression is absent undergo Archer multiplex fusion assay and FISH for ABL1, ABL2, PDGFRB, JAK2, and EPOR to assess for multitude of fusions that have been described in non-CRLF2 Ph-like ALL [62–64]. Unfortunately, the gold standard for assessing Ph-like ALL, namely gene expression profile, is not commercially available for routine assessment at this time. Ph-like ALL occurs in 25% of adult pre-B ALL. Eighty percent of Ph-like ALL have CRLF2 overexpression, 50% of them have *JAK* mutations (adverse; may still require allogeneic SCT in first CR), and 20% have ABL1 or PDGFR translocations and are treated on the Ph-positive ALL protocols (not reported here). The Ph-like alterations involving CRLF2/JAK delineate a high-risk B-ALL requiring intensified therapy and upfront allogeneic SCT [65, 66]. Patients with Ph-negative CRLF2/JAK Ph-like ALL have a disease that is more resistant to standard intensive chemotherapy, have high levels of persistent MRD positivity in CR and poor OS rates of 20–30%, and require more frequently allogeneic SCT to achieve long-term remission [54, 66]. Blinatumomab was shown to be effective in the relapse setting of B-ALL [15], improving the MRD negativity rate as well as survival [15, 67]. In a post hoc analysis from the phase III TOWER study, blinatumomab was effective in patients with and without Ph-like ALL, thereby negating the effect of Ph-like alterations [68]. The median OS was 7.9 months and 8.4 months, respectively, better than the survival observed with standard-of-care therapies [68]. The incorporation of blinatumomab into the frontline setting may negate the impact of the Ph-like phenotype through better MRD eradication, potentially eliminating the need for allogeneic SCT. At MD Anderson, we tested the efficacy of the frontline combination of Hyper-CVAD followed by blinatumomab and later (after 38 patients treated) the addition of lower-dose fractionated inotuzumab. The study aims were to improve efficacy, shorten the duration of intensive chemotherapy, and improve the treatment safety. With the amendment, we aimed to decrease the chemotherapy dose (inotuzumab given during even cycles with reduced-dose methotrexate at 500 mg/m^2^ on Day 1 and cytarabine 1 g/m^2^ every 12 h on Days 2–3), increase the rate of early MRD clearance by introducing inotuzumab, and improve survival [69, 70]. In the initial cohort, 38 patients (median age, 37 years) were treated with Hyper-CVAD plus sequential blinatumomab (4 courses, with later additional 3 courses, 1 every 3 months during POMP [prednisone, vincristine, methotrexate, 6-mercaptopurine] maintenance). High-risk features such as *TP53* mutation, *CRLF2* positivity, *KMT2A* rearrangement, and *JAK2* positivity were seen in 27%, 18%, 8%, and 5% of patients, respectively. The CR rate was 100% and the MRD negativity rate was 97%. The 3-year OS rate was 81%. Twenty-five patients (median age, 24 years) were treated after the amendment (which incorporated inotuzumab in Courses 2 and 4 of chemotherapy, and into 2 of the 4 later sequential blinatumomab courses); 15%, 15%, 10%, and 8% of patients had *TP53* mutation, *CRLF2* positivity, *JAK2* positivity, and *KMT2A* rearrangement, respectively. The CR rate was 100% and the MRD negativity rate was 91%. No relapses or deaths have occurred among patients who received inotuzumab, and the estimated 1-year OS was 100%. Overall, the CR rate was 100% in the entire cohort regardless of the Ph-like phenotype, and the MRD negativity rate was 95%, with no early deaths observed. The 3-year OS was 84%, with most patients completing the therapy within 1.5 years (compared to 3 years with conventional Hyper-CVAD followed by maintenance with POMP). The addition of antibodies to Hyper-CVAD chemotherapy seems to overcome the negative impact of high-risk disease features. Nevertheless, longer follow-up is needed to better evaluate the impact of this combination on preventing late relapses and maintaining long-term remission and survival (Fig. 3C). Other studies have also examined the role of adding blinatumomab to chemotherapy in patients with Ph-negative ALL, reporting similarly promising early results (Table 1) [71–73].

**Table 1:** Frontline blinatumomab and inotuzumab combinations in younger adults with newly diagnosed B-ALL

	Agent	*N*	Median age (range), years	Complete remission, %	MRD negativity, %	Overall survival, % (*x*-year)
Hyper-CVAD-blinatumomab [70]	Blinatumomab	38	37 (17–59)	100	97^**∆**^	81 (3-year)
Hyper-CVAD-blinatumomab-inotuzumab [70]	Blinatumomab and inotuzumab	25	24 (18–47)	100	91^**∆**^	100 (1-year)
GIMEMA LAL1913 [73]	Blinatumomab	149	41 (18–65)	90	96^**∆∆**^	84 (1-year)
GRAALL-2014-Quest [72]	Blinatumomab	95	35 (18–60)	NA	74^**∆∆**^	92 (1.5 year)
Low-intensity-blinatumomab [71]	Blinatumomab	30	52 (40–66)	100	83^**∆∆**^	69 (2-year)

*MRD* measurable residual disease

^∆^MRD negativity assessed by multicolor multiparameter flow cytometry at a sensitivity of 10^−4^

^∆∆^MRD negativity assessed by RT-PCR at a sensitivity of 10^−4^

### Older patients with B-ALL

Historically, older patients (≥ 55–60 years) with B-ALL have had poor outcomes with the standard intensive and dose-adjusted chemotherapy regimens, due a high incidence of adverse biologic features including adverse cytogenetics, *TP53* mutations [22, 74–77], and poor tolerance to intensive chemotherapy. The recent SEER ALL data showed a 5-year survival of 10–20% among patients 60 years and older [74]. The MD Anderson survival data for the same age group is shown in Fig. 3D. The improved survival from 20% in the era of chemotherapy to 40–50% since 2010 is due to the use of targeted agents, namely inotuzumab and blinatumomab, in combination with low-intensity chemotherapy in the frontline setting. The novel targeted therapies are less myelosuppressive (inotuzumab) and more active than conventional chemotherapy, hence the rationale for the combined modality approach in the frontline setting. Since 2010, we have investigated the induction and consolidation regimen with mini-Hyper-CVD (dose-reduced hyperfractionated cyclophosphamide, vincristine, dexamethasone) and inotuzumab +/ blinatumomab in older patients with newly diagnosed Ph-negative B cell ALL. The maintenance phase consisted of monthly courses of POMP for up to 3 years initially, which was later shortened after the amendment of the study to 12 courses of POMP and 4 courses of blinatumomab (1 course of blinatumomab after 3 courses of POMP) [78–80]. An interim analysis of 80 patients treated (median age, 68 years) showed an overall response rate (ORR) of 99% (CR rate 89%), an MRD negativity rate of 80% after the first course and of 94% at any time, with no early (4-week) mortality. The 5-year OS rate was 47%, 57% in patients aged 60–69 years, and 28% in patients 70 years and older. Six (8%) patients developed hepatic sinusoidal obstruction syndrome (SOS), of whom one after allogeneic SCT [81]. In a propensity score matching analysis, the mini-Hyper-CVD-inotuzumab +/ blinatumomab therapy showed a survival advantage compared with dose-adjusted Hyper-CVAD (3-year OS rate 63% versus 34%) [78]. Still, the mortality rate in remission with mini-Hyper-CVD-inotuzumab +/ blinatumomab was high in patients ≥ 70 years old compared with patients aged 60–69 years (70% versus 35%). This necessitated the modification of the study to reduce/eliminate the chemotherapy and shift toward a chemotherapy-free regimen in patients ≥ 70 years old (inotuzumab and blinatumomab).

The German Multicenter Study Group on ALL (GMALL) evaluated the use of induction inotuzumab followed by chemotherapy in 45 patients 55 years and older (median age, 64 years) [82]. The rate of CR or CR with incomplete count recovery (CRi) was 100%, the MRD negativity rate was 74%, and the estimated 2-year survival rate was 77%. One patient had suspected SOS [82]. The EWALL-INO study also showed the efficacy of induction therapy with inotuzumab in combination with low-intensity chemotherapy in 115 older patients (median age, 69 years). Among evaluable patients, the ORR was 86%, the MRD negativity rate was 73%, and the 1-year OS rate was 79% [83]. The Southwest Oncology Group (SWOG) 1318 reported on their experience in 29 older patients treated with frontline blinatumomab followed by POMP maintenance [84]. The CR rate was 66%, the 3-year OS rate was 37% (Table 2). Several randomized trials will assess the efficacy of chemotherapy-free regimens with drugs such as inotuzumab and blinatumomab (or with minimal chemotherapy) in older Ph-negative pre-B ALL.

**Table 2:** Frontline blinatumomab and inotuzumab combinations in newly diagnosed older B-ALL

	Agent	*N*	Median age (range), years	Complete remission, %	MRD negativity, %	Overall survival, % (*x*-year)
Mini-Hyper-CVD-Inotuzumab-blinatumomab [81]	Blinatumomab and inotuzumab	80	68 (60–87)	89	94^**∆**^	47 (5-year)
SWOG-1318 [84]	Blinatumomab	29	73 (66–86)	66	92^**∆**^	37 (3-year)
EWALL-INO [83]	Inotuzumab	115	69 (55–84)	79	73^**∆**^	79 (1-year)
GMALL Bold [144]	Blinatumomab	34	65 (56–76)	76	69^**∆∆**^	89 (1-year)
INITIAL-1 [82]	Inotuzumab	45	64 (56–80)	100	74^**∆∆**^	77 (2-year)

*MRD* measurable residual disease

^∆^MRD negativity assessed by multicolor multiparameter flow cytometry at a sensitivity of 10^−4^

^∆∆^MRD negativity assessed by RT-PCR at a sensitivity of 10^−4^

### Mature B cell leukemia/Burkitt leukemia

Burkitt leukemia is a rare subtype of B cell ALL. The use of rituximab in combination with Hyper-CVAD improved survival in Burkitt leukemia. This was confirmed in a randomized French trial [85, 86]. There has been a debate over whether Hyper-CVAD should remain the standard of care or be replaced by other less intensive regimens such as dose-adjusted EPOCH (DA-EPOCH; dose-adjusted etoposide, prednisone, vincristine, cyclophosphamide, doxorubicin, and rituximab). A study of 30 patients (median age, 33 years; age > 40 years, 40%) with Burkitt disease treated with either standard DA-EPOCH-R combination or lower-dose short-course EPOCH-R combination reported 7-year PFS and OS rates of 95–100% and 90–100%, respectively [87]. The majority of patients (90%) had low- and intermediate-risk disease. Recent data from a multicenter trial in 641 patients (median age, 47 years; range, 18 to 88 years) showed a promising outcome with DA-EPOCH [88]. The 3-year PFS and OS rates were 64% and 70%, respectively. In patients 40 years and younger, Hyper-CVAD therapy was more favorable than other regimens such as CODOX-M/IVAC (cyclophosphamide, doxorubicin, high-dose methotrexate/ifosfamide, etoposide, and high-dose cytarabine) and DA-EPOCH-R. However, in patients 60 years and older, Hyper-CVAD was associated with more toxicities [88, 89]. Patients with CNS disease and marrow involvement had less favorable outcomes and may require an intensive approach such as Hyper-CVAD. Burkitt cells express bright CD20, CD19 and CD22 positivity. Our current strategy in Burkitt leukemia incorporates blinatumomab and inotuzumab with R-Hyper-CVAD in patients < 60 years and with R-mini-Hyper-CVD in older patients.

### T cell ALL

T cell ALL is a unique separate ALL entity composed of different immunophenotypic and biologic subsets: thymic (CD1a +), mature (surface CD3 +), early (both negative) and its subcategory of early T cell precursor-ALL (ETP-ALL; myeloid markers positive, CD1a negative, CD8 negative, CD5 < 75%). At the cytogenetic and molecular level, ETP-ALL resembles acute myeloid leukemia (AML) or a hybrid ALL-AML, is associated with a poor prognosis, may benefit from AML-type therapies (hypomethylating agents and venetoclax), and benefits from allogeneic SCT in CR [90–93]. Effective anti-T cell targeted therapies have been lacking so far and immune therapies lagging behind for the management of T-ALL (in contrast to pre-B ALL). Therefore, improving the outcome in T cell ALL and T cell acute lymphoblastic lymphoma (T-LBL) has been more difficult and has relied on modifications of standard chemotherapies (high-dose cytarabine, high-dose methotrexate, asparaginase, nelarabine). Adding nelarabine, a prodrug of 9-β-arabinofuranosylguanine (ara-G), to chemotherapy in a Children’s Oncology Group (COG) trial improved event-free survival (EFS) in T-ALL but not in T-LBL [94]. In this study, the absolute benefit from the addition of nelarabine varied according to the associated methotrexate schedule. The 5-year DFS rates were 91% and 87% with escalating-dose methotrexate without leucovorin rescue plus pegaspargase with and without nelarabine, respectively; and 86% and 78% with high-dose methotrexate with leucovorin rescue with and without nelarabine, respectively [94].

At MD Anderson, we added nelarabine to Hyper-CVAD frontline therapy and combined it with asparaginase. This combination was associated with a 5-year OS of 60% [95]. In a retrospective analysis, a noticeable difference in outcome was seen in ETP-ALL versus other T-ALL [96]. A landmark analysis at 3 and 6 months showed that the addition of nelarabine to Hyper-CVAD improved survival (compared with Hyper-CVAD alone) in non-ETP T-ALL but not in T-LBL. “Near-ETP” ALL shares a similar transcriptional profile to ETP-ALL but with similar survival to non-ETP-ALL [96].

**Fig. 4:**
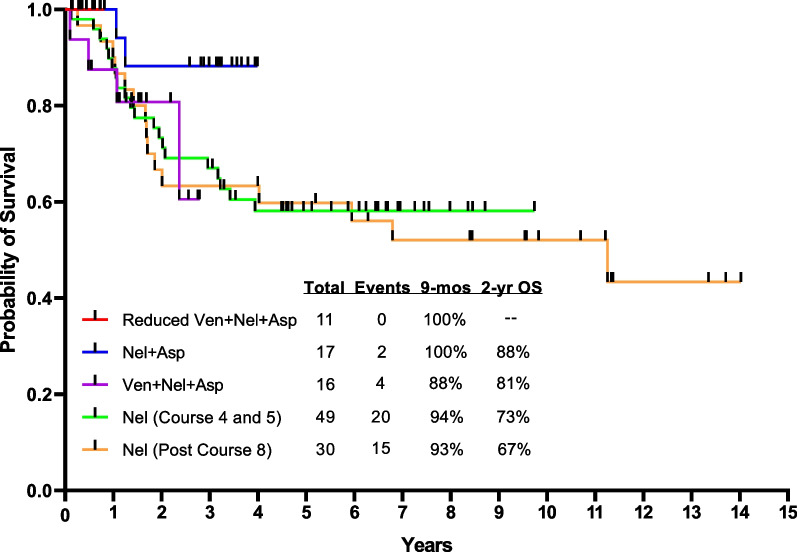
Overall survival of T-ALL by type of therapy. Abbreviations: asp, asparaginase; mos, months; OS, overall survival; nel, nelarabine; ven, venetoclax

Preclinical studies have shown that the BH3-mimetics, venetoclax and navitoclax, are active against B cell and T cell ALL, especially ETP-ALL [97–99]. Preliminary results of the combination of venetoclax with low-intensity chemotherapy in newly diagnosed older patients unfit for intensive chemotherapy showed objective response and MRD negativity rates of 91% and 100%, respectively [100]. At MD Anderson, venetoclax was added to frontline adult T cell ALL therapy with Hyper-CVAD-nelarabine-peg-asparaginase; 23% of patients enrolled on study had ETP-ALL. The ORR across all cohorts was 97%, with no early mortality. The median OS was 135 months (Fig. 4) [101]. The addition of venetoclax with dose adjustment might benefit selected patients.

Chimeric antigen receptor (CAR) T cell therapy is also being developed for the management of T-ALL. However, harnessing and redirecting normal T cells to recognize and destroy malignant T cells is associated with significant challenges due to the shared surface antigens, including T cell aplasia, fratricide and product contamination. Various strategies and solutions have been proposed to overcome these challenges. Early preclinical and clinical studies have shown an antitumor activity of CAR T cells against T cell malignancies, which have led to the conception of different phase I/II trials of CAR T cell products targeting CD4, CD5, CD7, CD30, and TRBC1 [102–104]. Recently, naturally selected CD7 CAR-T cells were shown to be safe and effective among 53 patients with T-ALL (*n* = 34) and T-LBL (*n* = 18) including those with extramedullary disease and a history of prior allogeneic SCT. CR with MRD negativity was seen in 96% of the patients and CRS ≤ Grade 2 in 89%; the 18-month OS rate was 75% [105].

### Prognostic impact of measurable residual disease

Measurable residual disease predicts outcome in ALL [106, 107]. Persistence of MRD at the time of morphologic remission confers a poor prognosis, unless addressed [108–113]. In a meta-analysis, undetectable MRD was associated with a better outcome, with a hazard ratio of 0.28 for EFS and OS [106]. This translated into 10-year OS rates of 60% with undetectable MRD, and 15% with persistent MRD. Different techniques are currently used for the evaluation of MRD in patients with ALL. RT-PCR is a highly sensitive assay that is broadly applied in Ph-positive ALL for the detection of MRD by quantifying *BCR::ABL1* mRNA transcripts. RT-PCR also allows for the quantification of MRD in Ph-negative ALL and T-ALL through allele-specific oligonucleotides able to detect unique sequences of the junctional regions of rearranged immunoglobulin IG or TCR genes [114]. So far, MRD in ALL has been assessed by multi-color flow cytometry (MFC) and RT-PCR assays [107]. We explored the role of NGS for the evaluation of MRD and compared the results from NGS with a sensitivity of 10^−6^, to MFC with a sensitivity of 10^−4^ [115]. Thirty-eight percent of patients with MFC-negative MRD were positive by NGS. Patients with negative MRD status by NGS at CR had 5-year cumulative incidence of relapse and survival rates of 13% and 100%, respectively. This supports the strategy of incorporating NGS-based MRD assessment in the frontline setting, along with MFC. In future practice, NGS should be used from the beginning for more accurate MRD assessment in order to tailor therapy and improve outcome in ALL.

### Therapeutic strategies for the eradication of measurable residual disease

In a European multicenter study, blinatumomab was evaluated among patients with B-ALL who achieve CR with persistent MRD. Among 113 evaluable patients, 88 (78%) converted to an MRD-negative status after one course of blinatumomab [67]. One hundred ten patients with Ph-negative ALL were evaluable for survival; 74 underwent allogeneic SCT. With a median follow-up of 53.1 months, the median OS was 36.5 months; the 4-year OS rate was 45%, 52% if MRD negativity was achieved. Continuous CR was noted in 40% of patients who underwent allogeneic SCT and 33% of patients who did not [116]. Favorable results were observed in our pilot study of 37 patients (median age, 43 years) with persistent MRD after first (73%) or second (27%) CR [117]. Patients received a median of 3 courses (range, 1 to 9 courses) of blinatumomab. Twenty-seven (73%) patients achieved complete MRD response within 2 courses of blinatumomab. Among the 19 patients with Ph-negative ALL, 83% achieved MRD negativity after a median of 41 days (range, 12 to 92 days). The overall 3-year OS rate was 67% [67, 116–118]. The improvement in outcome observed in our study compared with the European BLAST trial could be explained by several factors: 1) the threshold of disease burden for eligibility in our study was ≥ 1 × 10^−4^ compared with ≥ 1 × 10^−3^ in the BLAST trial. Early intervention with blinatumomab at levels < 1 × 10^−3^ was associated with favorable outcome with 3-year rates of relapse-free survival and OS of 83% and 77% versus 50% and 61% in patients treated with MRD ≥ 1 × 10^−3^. 2) In our study, patients received up to 5 blinatumomab courses (median 3 courses compared with 2 courses in the BLAST trial). 3) The flexibility for additional courses of blinatumomab for those unable to receive allogeneic SCT may have further improved the results.

Inotuzumab was also evaluated in 20 patients with MRD-positive pre-B ALL; 12 patients had Ph-positive ALL and 8 patients had Ph-negative ALL [119]. The median number of courses was 3 (range, 1 to 6 courses). Fourteen (70%) patients were in CR1, 11 (55%) had received prior blinatumomab and 4 (20%) had prior allogeneic SCT. MRD negativity was achieved in 12 (60%) patients, including 6 (75%) patients with Ph-negative ALL. One SOS occurred in this study [119].

### Relapsed/refractory ALL (Table 3)

Historically, R/R adult ALL carried a death sentence. With intensive chemotherapy, the CR rate was 30–50% and the median OS was 3–6 months. The 3-year survival rate was < 5–10%, even with allogeneic SCT in second CR [120, 121]. While inotuzumab and blinatumomab were both superior to standard of care chemotherapy and approved for the treatment of R/R B-ALL, the median survival was only 7.7 months with either modality, and the long-term (at 2–3 years) OS was 25% or less [15, 16]. At MD Anderson, we have investigated since 2010 a combination of low-intensity mini-Hyper-CVD chemotherapy with targeted therapies. Inotuzumab was added to mini-Hyper-CVD on Day 3 of each of the first 4 courses at 1.8 to 1.3 mg/m^2^ for Course 1, followed by 1.3 to 1.0 mg/m^2^ for subsequent courses. The protocol was later amended to lower and fractionate the inotuzumab dose into weekly doses (0.6 and 0.3 mg/m^2^ during Course 1 and 0.3 and 0.3 mg/m^2^ during subsequent courses) and to add sequential blinatumomab [122–124]. A total of 112 patients (67 without blinatumomab and 45 with blinatumomab) were treated; 71% were treated in Salvage 1 and 29% in Salvage 2 +. Overall, 93 (83%) patients responded, 70 (63%) of whom achieved CR. The overall MRD negativity rate among responders was 84%. Fifty-three (47%) patients were able to proceed to allogeneic SCT. The median OS was 17 months, significantly superior to historical results with inotuzumab. The 3-year OS rate was 41%. The addition of blinatumomab to mini-Hyper-CVD-inotuzumab in a sequential fashion, along with a lower dose of inotuzumab, improved outcomes. The median OS and 3-year OS were 14 months and 34% pre-amendment, compared with 37 months and 55% post-amendment, respectively. Better outcome was seen in Salvage 1 compared with Salvage 2 +, with 3-year OS rates of 51% versus 17%. No difference in survival was seen according to allogeneic SCT status in patients treated with this regimen. The median OS was 47 months, and 3-year OS rate was 55% in patients who underwent allogeneic SCT, compared with 31 months and 48% in those who did not, respectively. The rate of SOS also improved from 13% before the amendment to 2% after the amendment. These findings highlight the improved safety and efficacy with the addition of blinatumomab to mini-Hyper-CVD and with inotuzumab administered at lower weekly doses (together with ursodiol SOS prophylaxis). However, patients who fail to respond to blinatumomab- and inotuzumab-based combinations have dismal outcomes, with limited treatment options [125]. As blinatumomab and inotuzumab are being incorporated into the frontline setting, novel treatment strategies are needed for R/R patients such as CAR T cell therapies, venetoclax-based combinations, or other investigational drugs.

**Table 3:** Relapsed/refractory Ph-negative ALL

Study	Agent	*N*	Median age (range), years	Complete remission, %	MRD negativity, %	Overall survival, % (*x*-year)/Median (months)
INO-VATE [151]	Inotuzumab	164	47 (18–78)	74	71^**∆**^	20.3 (3-year)/7.7
TOWER [15]	Blinatumomab	271	41 (18–80)	34	76^**∆∆**^	NA/7.7
ELIANA [129]	Tisagenlecleucel	97*	11 (3–24)	66	98^**∆**^	55** (5-year)/NA
KTE-X19 [131]	Brexucabtagene autoleucel	71***	40 (28–52)	44	97^**∆**^	NA/25.4
MDACC [124]	Mini-Hyper-CVD-Inotuzumab	67	34 (17–87)	60	82^**∆**^	34 (3-year)/14
MDACC [123]	Mini-Hyper-CVD-Inotuzumab-Blinatumomab	45	42 (18–79)	67	85^**∆**^	55 (3-year)/37

*MRD* measurable residual disease, *NA* not available

^∆^MRD negativity assessed by multicolor multiparameter flow cytometry at a sensitivity of 10^−4^

^∆∆^MRD negativity assessed by either multicolor multiparameter flow cytometry or RT-PCR at a sensitivity of 10^−4^

*79 infused; **among 66 patients in complete remission; ***55 infused

We are exploring the value of sequential CAR T cell therapies delivered at the time of CR1 or CR2. Currently, two CD19-directed CAR T cell products are approved for the management of ALL, tisagenlecleucel (Salvage 2 +; age < 26 years) and brexucabtagene autoleucel (R/R disease irrespective of salvage status; age ≥ 18 years) [126, 127]. Among 97 patients (age < 26 years) enrolled on the ELIANA (tisagenlecleucel) trial, 65 (67%) achieved CR. Grade 3–4 cytokine release syndrome (CRS) was reported in 49% [128]. In a recent update, the 5-year EFS and OS rates among 66 patients treated with tisagenlecleucel were 42% and 55%, respectively [129]. The presence of detectable MRD by NGS at 3 months after tisagenlecleucel therapy independently predicted for worse EFS and OS by multivariate analysis [130]. Brexucabtagene autoleucel therapy (71 patients; median age 40 years; range, 28 to 52 years) resulted in an ORR of 55%, an MRD negativity of 97% among responders, and a median OS of 18.2 months. Grade ≥ 3 CRS was seen in 24% [127]. In a recent update, the median overall survival was 25.4 months among evaluable patients, with better survival observed in patients with lower disease burden [131]. Park and colleagues also reported better outcomes when CAR T cell therapy was offered to adult patients with low disease burden (< 5% bone marrow blasts; median EFS 10.6 months; median OS 20.1 months) compared to those with higher disease burden (≥ 5% bone marrow blasts or extramedullary disease; median OS 12.4 months; estimated 2-year OS around 10%) [132]. The Real World CAR Consortium data showed that the best outcome with CAR T cell therapy was in patients receiving therapy in low disease burden or no detectable disease, compared with high disease burden [133]. Despite the high remission rates observed with CD19 CAR T cells, relapses eventually occur in more than 50% of the patients, primarily driven by the downregulation or the loss of CD19 surface antigen expression [134, 135]. CD22 is expressed on most B-ALL leukemic cells and is usually retained following CD19 loss. In a phase I trial, 21 children and young adults (17 of whom previously exposed to CD19 CAR T cells) were treated with CD22-targeted CAR T cells. Results showed a dose-dependent antileukemic activity, with a CR rate of 73% in patients receiving ≥ 1 × 10^6^/kg CD22 CAR T cells [136]. Another CD22 CAR T cell product was evaluated in a pediatric and adult population and resulted in a CR rate of 75% and MRD negativity of 56% [137]. Similar findings were observed with a novel CD22 CAR T cells product evaluated in 19 heavily pretreated children and young adults relapsing with CD19-negative B-ALL after treatment with CD19 CAR T cells. Of 17 patients infused, 13 (77%) achieved CR and 10 (59%) MRD negativity at day 28 of the infusion. A bispecific CAR product targeting CD19 and/or CD22 (CD19-22.BB.z-CAR) is also being evaluated in a phase I trial of patients with R/R B cell malignancies. Among 17 patients with R/R B-ALL (50% of relapses had low or absent CD19 expression), the ORR and MRD negativity rates were 88% and 100%, respectively [135]. Based on these observations, immunotherapy approaches including antibody–drug conjugates, BiTEs, and CAR T cell therapy, are complementary. We are currently exploring a consolidative approach with dose-dense mini-HCVD-inotuzumab-blinatumomab followed by CD19 CAR T cells.

### Management of CNS relapses

The incidence of CNS leukemia in adults with ALL is around 5%–10% at diagnosis and 5% at relapse using current standard therapies [138]. Patients with CNS recurrence have a poor outcome, with an historical survival of less than 1 year [139]. The anti-CD19 and CD22 targeted therapies, blinatumomab and inotuzumab, do not cross the blood–brain barrier, and therefore, they are not recommended for treating active CNS relapses [140, 141]. Treatment with CAR T cells has demonstrated its efficacy in patients with B-ALL and CNS relapses with or without bone marrow disease. In a retrospective analysis of 48 patients with R/R B-ALL, the administration of CD19 CAR T cells resulted in a remission rate of 85% in the CNS, and a cumulative incidence of CNS relapse of 11% at 12 months. Grade 3–4 neurotoxicity was reported in 23% of patients, with a higher frequency in those with a higher CNS disease burden before the CAR T cells infusion [142]. Preliminary findings from a post hoc analysis of pooled data from five clinical trials also support the use of CAR T cells in patients with CNS relapses. Treatment with tisagenlecleucel and huCART19 in 66 patients with CNS relapses resulted in a CR rate of 97% at Day 28; however, 42% of responders relapsed again in the CNS after CAR T cell infusion. The incidence of Grade 3–4 neurotoxicity and CRS was 11% and 29%, respectively [143].

## Conclusions

In this analysis, we report our comprehensive data results from 1985 to 2022 in adults with ALL treated at our institution. Significant improvements have been documented in all subtypes of ALL since 2000 and 2010.

In Ph-positive ALL, therapy has shifted from intensive chemotherapy and allogeneic SCT before 2000 to regimens combining BCR::ABL1 TKIs with intensive or lower intensity chemotherapies followed by allogeneic SCT (2000–2010), the addition of ponatinib to intensive chemotherapy (2010–2018) demonstrating for the first time that allogeneic SCT may not be a requirement for a better cure, and finally the recent shift to (mostly) non-chemotherapy non-SCT targeted strategies with BCR::ABL1 TKIs and blinatumomab.

Similar improvements in outcomes were observed in younger patients using Hyper-CVAD in combination with antibodies targeting CD20 (rituximab, ofatumumab), CD19 (blinatumomab), and CD22 (inotuzumab). The recent trials incorporating blinatumomab and inotuzumab were associated with estimated 3-year OS rates above 80%. In older B-ALL, less intensive chemotherapy in combination with inotuzumab and blinatumomab almost doubled the 5-year survival rate (50% versus 25% with historical data of dose-adjusted Hyper-CVAD). Patients ≥ 70 years still have a poor outcome attributed to deaths in CR and the development of myelodysplastic syndrome and AML. In them, combinations of blinatumomab and inotuzumab with minimal chemotherapy might help [71–73, 82, 84, 144].

The benefit observed with the addition of nelarabine in T-ALL is in line with the findings of the Children's Oncology Group (COG) trial, where the addition of nelarabine in non-ETP and non-lymphoblastic lymphoma has shown a survival benefit [94]. In the last update, further addition of asparaginase to the Hyper-CVAD regimen is favorable as well and may negate the poor baseline biological features and spare the need for allogeneic SCT as reported by Pui and colleagues (St Jude Total therapy XV) [145] and the Acute Leukemia Committee of the CIBMTR and the Dana Farber ALL Consortium, where younger adults (median age, 30 years; range, 18 to 50 years) with Ph-negative ALL treated with asparaginase-based therapy had lower treatment-related mortality rates, less relapses, and superior OS compared to allogeneic SCT [146]. Through BH3 profiling, Chongaile et al. have shown that ETP-ALL is BCL-2 dependent and is very sensitive to in vitro and in vivo treatment with venetoclax [147]. Furthermore, the combination of venetoclax, with low-dose navitoclax, a BCL-XL/BCL-2 inhibitor, and chemotherapy, was well tolerated and had promising efficacy in patients with relapsed/refractory disease [148]. Venetoclax with or without navitoclax combined with Hyper-CVAD (NCT03319901) or mini-Hyper-CVD (NCT03808610) are being evaluated in frontline T-ALL and relapsed/refractory Ph-negative ALL. Finally, the inhibition of LCK pathways may lead to a better outcome as well, and this has been shown in vitro where the LCK pathway activation was the driver of dasatinib sensitivity in some of the patients with T-ALL [149]. Currently, we are exploring the role of ponatinib in combination with mini-Hyper-CVD and venetoclax in T-ALL (NCT05268003).

The use of NGS for MRD assessment is superior to conventional MRD assays. Similar to the pediatric experience [150], we have shown that patients who achieve early MRD negativity by NGS have the best survival. No relapses were seen in our analysis; however, a longer follow-up with a larger cohort of patients is needed to confirm these findings. If confirmed, then the speed and depth of NGS response may help in the selection of the kinds of therapies needed and tailor the duration of therapy based on the duration of NGS MRD negative status. In Ph-positive ALL, the assessment of NGS MRD early on may allow designing trials that explore TKIs discontinuation, as is now practiced in chronic myeloid leukemia.

Novel bi-specific, tri-specific, and tetra-specific T cell engagers targeting CD19, CD20 and CD22 are under development. So far, CAR T cells have been assessed in the R/R B-ALL population, but their optimal use may be in the setting of minimal disease. Future trials at MD Anderson will assess the role of CAR T cells as a consolidation in frontline high-risk ALL and in second or later CRs. The MRD assessment by NGS post-CAR T cells will help identify patients who may not need allogeneic SCT.

In conclusion, the management of ALL is in the midst of a slow-motion therapeutic revolution. Novel combinations including second–third-generation BCR::ABL1 and novel antibodies are questioning the need and duration of intensive chemotherapy and allogeneic SCT. With these approaches, the outcomes in adult ALL may become potentially as favorable as those observed in pediatric ALL. Perhaps within the next 5 to 10 years, adult ALL therapy may change from the current prolonged intensive chemotherapy of 2–3 years to shorter and less intensive chemotherapy regimens (and perhaps no chemotherapy in Ph-positive and elderly ALL subsets) in combination with TKIs, targeted therapies directed at CD19, CD20 and CD22, BCL-2 inhibitors, and CAR T cells. While randomized controlled trials are useful, they may be outdated by the time the data mature, and this could represent an obstacle for the approval of new drugs. Rather, Bayesian design studies and prospective trials are encouraged. This strategy can help accelerate the development of new therapeutic strategies which might have the potential of improving disease outcomes.

## Data Availability

The datasets used and/or analyzed during the current study are available from the corresponding author on reasonable request.

## Abbreviations

ALL: Acute lymphoblastic leukemia
AML: Acute myeloid leukemia
AYA: Adolescent and young adults
BiTE: Bispecific T cell engager
CAR: Chimeric antigen receptor
CMR: Complete molecular response
CNS: Central nervous system
COG: Children’s Oncology Group
CR: Complete remission
CRi: Complete remission with incomplete count recovery
CRLF2: Cytokine receptor-like factor 2
CRS: Cytokine release syndrome
EFS: Event-free survival
ETP-ALL: Early T cell precursor-ALL
GMALL: German Multicenter Study Group on ALL
IT: Intrathecal
MFC: Multi-color flow cytometry
MRD: Measurable residual disease
NGS: Next-generation sequencing
ORR: Overall response rate
OS: Overall survival
Ph: Philadelphia chromosome
PFS: Progression-free survival
R/R: Relapsed/refractory
SCT: Stem cell transplantation
SEER: Surveillance, Epidemiology, and End Results
SOS: Sinusoidal Obstruction Syndrome
SWOG: Southwest Oncology Group
T-LBL: T cell acute lymphoblastic lymphoma
TKI: Tyrosine kinase inhibitor
WHO: World Health Organization
